# Book Reviews

**Published:** 1897-09

**Authors:** 


### BOOK REVIEWS.

A System of Diseases of the Eye. By American, British, Dutch, French, German and Spanish authors. Edited by William F. Norris, A.M., M.D., and Charles A. Oliver, A.M., M.D., Philadelphia. In four volumes. *Vol. II. Examination of the Eye, School Hygiene, Statistics of Blindness and Antisepsis.* Published by J. B. Lippincott Company, Philadelphia. Price, $5.

In the second volume of this welcome system the contributors are all Americans with the exception of Messrs. Snellen of Utrecht, Laqueur of Strassburg, Javall of Paris, and Wilbrand of Hamburg. For the section on the Methods of Determining the Acuteness of Vision, the editors have had the fortune to secure the services of Professor Herman Snellen of Utrecht, with Mr. Berry of Edinburgh, as translator. Though the paper is short it contains an ample discussion of the various tests, the influence of illumination, age, refractive errors, etc. Herman Snellen, Jr., contributes a short but sufficient account of the various mydriatics and myotics. Laqueur's paper on lateral illumination is translated by Harry Friedenwald of Baltimore, and, if somewhat elementary, is yet likely to prove useful to many who have not yet discovered the value of this aid to diagnosis. In his contribution on the Ophthalmoscope, Dr. George Gould, of Philadelphia, has given a short history of the instrument, has explained its physics, and has pictured and described several of the most valuable instruments in use. The interesting fact is here mentioned that Mr. Babbage showed Professor Wharton Jones an ophthalmoscope of his own invention four years before Helmholz produced his, and that it was, moreover, of a much more advanced type than Helmholz's, being a plane mirror with the silver scraped off at the center.

Dr. Jackson, of Philadelphia, writes on Skiascopy, a subject which he has made very much his own, and the younger Javal deals with the ophthalmometer and its clinical applications, an instrument adopted in America much more kindly than in any European country, with results to American ophthalmology which are certainly not wholly advantageous, especially to the younger men. Javal discusses the observations related in the "immortal memoir" of Thomas Young, describes the ophthalmometer of Helmholz, and then proceeds to a detailed description of that of Javal-Schiötz, as found in the different models which have been produced in Paris. The paper includes an account of the practical applications of this instrument as applied to measurements of the curves of the anterior and posterior surfaces of the cornea, and of the anterior surface of the lens. It is to be regretted that Dr. Javal has omitted even to mention the ophthalmometer of Reid, of Glasgow, which has several advantages over that of Javal and Schiötz, one of which is its portability.

The section on Prisms has been undertaken by Dr. W. S. Dennet, of New York. This, for a short treatise, contains more mathematics than will be agreeable to most readers, but their practical application is not forgotten. In the paper entitled "The Principles of and the Methods for the Estimation of the Balance of the Extra-Ocular Muscles," Dr. Stevens, of New York, takes up a subject with which his name is closely associated. He classifies the various anomalies of "balance" of these muscles and describes the different methods which have been devised for their measurement, but does not here undertake the subject of their treatment.

One of the most complete, and a very valuable part of the book, is that by Dr. Herman Wilbrand, of Hamburg, Germany, on Perimetry and its Clinical Value. In the 126 pages which this treatise occupies, the author deals in a remarkably able manner with the various forms which the field of vision may assume, and connects these with their different causes. He gives seventy-seven charts and one colored plate of Henschen's scheme of the visual paths. Dr. Thomas Pooley is the translator, and he has rendered the German into an agreeable English, not always an easy task. To Dr. William Thomson, assisted by Dr. Carl Weiland, both of Philadelphia, has been entrusted the department dealing with the Detection of Color Blindness. Dr. Thomson was already well known in this connection and this present work cannot but enhance the reputation of himself and his assistant. How carefully Dr. Risley, of Philadelphia, has gone into his section of the work will be seen at a glance. "School Hygiene" is a subject, which, like every other in the department of preventive medicine, has been too long neglected, and Dr. Risley's excellent article cannot but help to quicken into new life the attention of all who care for the proper and healthy development of the present and future of our kind. It is well worth perusal by those of influence outside the walls of the profession.

Dr. Minis Hays, of Philadelphia, has written on "Blindness: Its Frequency, Causes and Prevention." His paper is of necessity to a considerable extent statistical; it considers other countries besides this, and takes up the relationship of climate, age, sex and occupation to blindness; goes into its causes as they are found in the different subdivisions of the eye, as well as the best method of prevention. Dr. Joseph Andrews has written a useful article on Antisepsis, and Drs. J. McFarland and S. S. Kneass, of Philadelphia, have together contributed the section on the "Micro-organisms of the Conjunctiva and Lacrymal Sac."

There is a good index, the type is distinct, and the publishers have produced a handsome volume. The book is one of great value and will well repay its purchasers. A. W. S.

---

A SYSTEM OF PRACTICAL MEDICINE. By American authors. Edited by Alfred Lee Loomis, M.D., late Professor of Pathology and Practical Medicine in the New York University, and William Gilman Thompson, M.D., Professor of Materia Medica, Therapeutics and Clinical Medicine in the New York University. In four volumes. Volume II. Diseases of the Respiratory and Circulating Systems, and of the Blood, Kidneys and Genito-Urinary Organs. Price per volume, cloth, $5.00. Lea Bros. & Co., Publishers, Philadelphia and New York.

The first volume of this elegant work was noticed in our March number. The second equally deserves the highest commendation. The contributors to this volume are Drs. R. C. Cabot, of Boston; Thos. D. Coleman, of Augusta, Ga.; Warren Coleman, of New York; E. G. Cutler, of Boston; I. N. Danforth, of Chicago; R. H. Fitz, of Boston; W. W. Gannett, of Boston; Irving S. Haynes, of New York; Alfred L. Loomis, of New York; Henry P. Loomis, of New York; A. Lawrence Mason, of Boston; Chas. E. Quimby, of

New York; F. C. Shattuck, of Boston; S. Edwin Solly, of Colorado Springs; James Tyson, of Philadelphia; H. B. Whitney, of Denver, and Jas. T. Whittaker, of Cincinnati. The volume includes Diseases of the Respiratory System (excepting tuberculosis), Diseases of the Circulating System and Mediastinum, Diseases of the Blood, Diseases of the Kidneys, Diseases of the Bladder and Prostate Gland, Abnormalities of the Urine, and Uremia. The volume contains seventy-four illustrations and fifteen half-tone plates well executed. The lamented Professor Loomis contributes the chapter on Endocarditis in the plain and forcible style which distinguished all his writings. (This we understand was the last literary work of Dr. Loomis.) The sections on Diseases of the Blood and Bright's Disease are particularly complete and up to date. We are pleased to notice among the contributors Dr. Coleman, of Augusta, who furnishes the sections on Uremia and Abnormalities of the Urine.

All the articles in this system have been written by good men, eminently qualified for their respective tasks, and each has presented his subject in a manner which will not fail to satisfy every reader.

---

A TEXT-BOOK OF DISEASES OF WOMEN. By Charles B. Penrose, M.D., Ph.D., Professor of Gynecology in the University of Pennsylvania; Surgeon to the Gynecean Hospital, Philadelphia. W. B. Saunders, Publisher, Philadelphia. $3.50 net.

As the author states, this book was written for the medical student. It is a volume of about five hundred pages, of good mechanical execution. The drawings or cuts serve to illustrate the purpose for which they were intended, though some of them are a little stiff and ungraceful. The text is necessarily condensed that general principles might be presented without useless reading by the student who usually has as much of it to do as the average mind can appropriate in the short time allowed by the curricula of our medical institutions. Unfortunately it is, perhaps, a little too brief in some of its subjects, for instance, pathology, histology and bacteriology—relying mainly upon macroscopical rather than microscopical appearances of diseased tissue. Again, some details in plastic operations are not quite as clear as the beginner should have them presented to him, but on the whole the book is a good one and contains much more than the student or general practitioner will master in connection with their other labors.

While a gynecologist would regret the brevity or condensed form of this work, the medical student will find it a very happy volume for his purposes—aiming directly at practical, clinical gynecology as it does. N.

---

REFERENCE BOOK OF PRACTICAL THERAPEUTICS. By various authors. Edited by Frank P. Foster, M.D. Vol. II. Appleton & Co., New York.

Since reviewing volume I. of this book we have had ample occasion to test it as a practical guide, especially in its relationship to the more recent drugs, and without disappointment. Their alphabetical arrangement and the heavy type of the heading facilitate reference, and without being too diffuse the matter itself contains a full account of the various properties of each drug, the therapeutic actions being especially carefully considered. The second volume follows exactly in the footsteps of the first, and will be an equally valuable addition to the library of those who desire to be abreast of the times in this most important branch of medicine. The contributors to this volume are Drs. Armstrong, Brickner, Bronson, Coley, Crandall, Foster, Gerster, Griffin, Lilienthal, Peabody, Peterson and Rice, all of New York; Drs. Eskridge, of Denver; Jewett, of Brooklyn; Potter, of San Francisco; Solis-Cohen, of Philadelphia; and Whittaker, of Cincinnati.

We recommend this book with much satisfaction, in the assurance that its possessors will find it of much value. A. W. S.

---

SURGERY OF THE RECTUM AND PELVIS. By Chas. B. Kelsey, A.M., M.D., New York, Professor of Surgery at New York Post-Graduate Medical School and Hospital. With two hundred and eighty-one illustrations and half-tone plates. Richard Kettles & Co., New York.

Dr. Kelsey's work on Diseases of the Rectum is too well known to require more than a passing notice at this time. An exceptional experience in the use of the clamp and cautery operation for hemorrhoids has increased, if possible, his faith; he unhesitatingly pronounces it the best operation for internal hemorrhoids. He practically condemns the Whitehead operation. His condemnation of the injection method of treating hemorrhoids is less severe than in former editions, and amounts to almost a recommendation of its use in some cases of internal hemorrhoids. This division of the work embraces the well known views of the author as expressed in former publications. The various diseases of the male and female pelvic organs are also considered. Many of the operations on these organs are clearly described by concise text and well-chosen illustrations. Hernia, umbilical, ventral, inguinal and femoral, are also discussed concisely. Intestinal anastomosis and appendicitis also receive a share of the author's consideration. This grouping of subjects is a departure in medical literature. In a work of only 554 pages of reading-matter, they must necessarily be treated in a very cursory manner, and the wisdom of such a procedure, it seems to me, is very questionable. F. W. M.

---

Essays on Social Topics. By Lady Cook (*née* Tennessee Claflin). Published by The Roxburgh Press, 15 Victoria street, Westminster, London.

The contents of this little book are somewhat novel, as regards both the subjects treated and the views expressed. The subjects, however, are live and the essays lively and vigorous. Among these are Virtue, Maternity, Prudery, Regeneration of Society, Marriage, Wrongs of Married Men, Wives and Mistresses, Degradation of the Sexes, Illegitimacy, Who Should Propose?, One of the Evils of Society, Should the Poor Marry, Woman's Purity, Advice to Parents, Mothers and Their Duties, History of Marriage, etc., etc. Lady Cook believes that the women "should have the privilege of proposing," and that "society should discourage the remarrying of widows." While the views expressed by the author upon several of these topics may be somewhat advanced, it is very evident that she is terribly in earnest. Spades are spades here, and there is not a dull page in the book.

